# Neural correlates of lateral modulation and perceptual filling-in in center-surround radial sinusoidal gratings: an fMRI study

**DOI:** 10.1038/s41598-022-20592-y

**Published:** 2022-09-27

**Authors:** Yih-Shiuan Lin, Chien-Chung Chen, Mark W. Greenlee

**Affiliations:** 1 Institute of Experimental Psychology, University of Regensburg, Universitätsstraße 31, 93053 Regensburg, Germany; 2 Department of Psychology, National Taiwan University, Taipei, Taiwan; 3 Neurobiology and Cognitive Science Center, National Taiwan University, Taipei, Taiwan

**Keywords:** Pattern vision, Human behaviour, Striate cortex

## Abstract

We investigated lateral modulation effects with functional magnetic resonance imaging. We presented radial sinusoidal gratings in random sequence: a scotoma grating with two arc-shaped blank regions (scotomata) in the periphery, one in the left and one in the right visual field, a center grating containing pattern only in the scotoma regions, and a full-field grating where the pattern occupied the whole screen. On each trial, one of the three gratings flickered in counterphase for 10 s, followed by a blank period. Observers were instructed to perform a fixation task and report whether filling-in was experienced during the scotoma condition. The results showed that the blood-oxygen-level-dependent signal was reduced in areas corresponding to the scotoma regions in the full-field compared to the center condition in V1 to V3 areas, indicating a lateral inhibition effect when the surround was added to the center pattern. The univariate analysis results showed no difference between the filling-in and no-filling-in trials. However, multivariate pattern analysis results showed that classifiers trained on activation pattern in V1 to V3 could differentiate between filling-in and no-filling-in trials, suggesting that the neural activation pattern in visual cortex correlated with the subjective percept.

## Introduction

It is well known that a surround pattern can affect the percept of a central stimulus^1–3^. In visual crowding, for example, the presence of flankers diminishes the recognition of the center target^4–6^. In the lateral masking paradigm, lateral flankers first increase then decrease the detection threshold of a central target as the distance between the target and flankers increased^7^. In the presence of a superimposed pedestal, colinear lateral flankers decrease the target discrimination threshold at low pedestal contrast and increase the threshold at high pedestal contrast^8^. In visual illusions, such as the Ebbinghaus illusion and tilt illusion, the size or orientation percept of a central target can be modulated by its surround^2,9–11^. Numerous studies have reported that these phenomena can result from facilitation and suppression arising from the surround and these effects depend on the stimulus eccentricity, luminance contrast, orientations and other features of such flanking stimuli^8,12–21^. These results imply that neurons respond to different parts of the visual field and interact with each other in a complex fashion.

Perceptual filling-in, in which the visual system compensates for missing information in a region by interpolating information of the surrounding features, is yet another demonstration of the lateral modulation effect^22–25^. Filling-in can be observed under numerous viewing conditions. For example, with monocular viewing we do not perceive a black hole at the location of the blind spot, where there are no photoreceptors due to the optical nerves passing through. The absence of awareness of the blindspot is related to filling-in of the surround texture or color^26–28^. Filling-in also happens in the retinal scotoma of patients with eye diseases such as macular degeneration^29–31^. A further scenario where filling-in occurs is when an artificial scotoma is induced, where a blank region embedded among dynamic noise or texture background disappears after prolonged steady fixation^23,32–34^. Filling-in can also be observed in certain visual illusions. In water-color illusion, color is perceived to spread to the white space in between two-toned colored wiggly stripes^35^. Similarly, in neon-color illusion, illusory color is perceived and spreads out to an illusory border^36^. Another example is the Troxler’s fading effect, in which following steady fixation, a ring with blurred edges fades away from sight^37^. In Craik–O'Brien–Cornsweet illusion^38^, the perceived brightness level of a luminance surface is increased or decreased depending on the edge as the perceived brightness of the edge spreads out to the whole surface.

Behavioral and neuroimaging studies on perceptual filling-in have focused on the latency and properties of different types of filling-in effects as well as on the underlying mechanisms (see^39^ and^40^ for a review). However, the exact neural processes involved in the filling-in percept are still under debate. Two theories have emerged to explain the neural mechanisms of filling-in. The first, the cognitive or symbolic theory, suggests that filling-in results when the visual system ignores the scotoma regions and thus involves no active processing^41,42^. The second, the isomorphic theory, states that a spread of neural activation occurs from the border to the center of the filled-in region, pointing to an active process^32,43^. While both theories have their merits and can explain most of the filling-in phenomena, empirical results from neurophysiological and neuroimaging studies have shown that visual areas are activated during filling-in, supporting the isomorphic theory. For instance, Murakami^44^ demonstrated that after the blind spot region of one eye was being adapted to a drifting grating, participants experienced a motion aftereffect in the corresponding region of the other eye, indicating that the filled-in motion in the blind spot activated a direction-selective neural mechanism during adaptation. In a single-neuron recording experiment, Matsumoto and Komatsu^27^ detected increased neural activity in macaque V1 when filling-in occurred in the blind spot (see also^45^). De Weerd et al.^32^ also discovered that neurons with receptive fields inside an artificial scotoma were activated when filling-in was perceived. More evidence of the involvement of active processing has been reported in Craik–O'Brien–Cornsweet illusion with electrophysiological recordings^46^ and in neon-color spreading with functional magnetic resonance imaging (fMRI)^47^.

In addition to results showing that the filled-in region is related to lateral excitation from the surround, other studies have shown evidence for lateral inhibition from the surround region during filling-in. Chen et al.^48^ presented flickering high-contrast checkerboard pinwheels to the participants in an MRI scanner and discovered that visual cortex corresponding to the unstimulated inter-wedge regions showed decreased BOLD (Blood-oxygen-level-dependent) activation. Participants reported experiencing a twinkle aftereffect in the unstimulated regions after the pinwheel stimulus disappeared, a common illusory percept observed in artificial scotoma after a surround texture is removed^33,49–52^. Such a “rebound” effect suggests a release from surround suppression of the unstimulated regions after the surround stimulus disappeared. Supèr and Romeo^53^ proposed a computational model in which surround inhibition creates rebound in neurons in the unstimulated regions to explain filling-in. In a later fMRI study, adapting the lateral flanker paradigm, Chen^54^ was able to partition two inhibitory components from the surround suppression in BOLD signals: an orientation-specific inhibition that happened when the central target and flankers were colinear, and a general inhibition that occurred whenever flankers were present regardless of their orientations. Weil et al.^55^ demonstrated a reduced MEG (magnetoencephalography) power when the target was being filled in. In a subsequent fMRI study^56^, the same group showed decreased BOLD signals in V1 and V2 when filling-in was perceived. Mendola et al.^57^ reported reduced brain activation in V1 and V2 and enhanced activation in V3 and higher regions during filling-in. Behavioral studies also showed similar lateral inhibition effects in filling-in. Mihaylov et al.^58^ reported elevated detection threshold of a Gabor target placed inside an artificial scotoma surrounded by dynamic noise only when the surround texture induced filling-in and twinkle aftereffect, suggesting that the lateral modulation involved in filling-in contribute to the sensitivity reduction.

The fact that evidence for both lateral excitation and lateral inhibition have been shown reveals the complexity of the underlying neural mechanism and the possibility that more than one process is involved. More research is therefore needed to collect further empirical evidence of the neural correlates of perceptual filling-in.

Previously, in a psychophysics study^16^, we selectively adapted the center, surround, or both the center and surround regions in the periphery with oriented-sinusoidal gratings and estimated the tilt-aftereffect (TAE) induced in a subsequent Gabor target located in the center region. Perceptual filling-in was observed during the surround-only adapter presentation, where the central hole represented an artificial scotoma. Our results showed that the TAE magnitude was strongest in the center-only condition, intermediate in the center plus surround condition, and it was weakest in the surround-only condition. The reduction of TAE in the center-surround compared to the center-only condition indicated an inhibitory lateral modulation effect when a surround was added to the adapter center. We also discovered that such lateral inhibition was orientation-specific^17^. In terms of the surround-only condition, we found a positive correlation between the subjective filling-in percept and the TAE magnitude. Additionally, when we separately estimated the TAE on trials with and without filling-in, the adaptation effect was stronger in the former, suggesting that filling-in was associated with a more pronounced lateral modulation effect. Our behavioral results revealed clear evidence of lateral modulation effect involved in perceptual filling-in.

**Figure 1:**
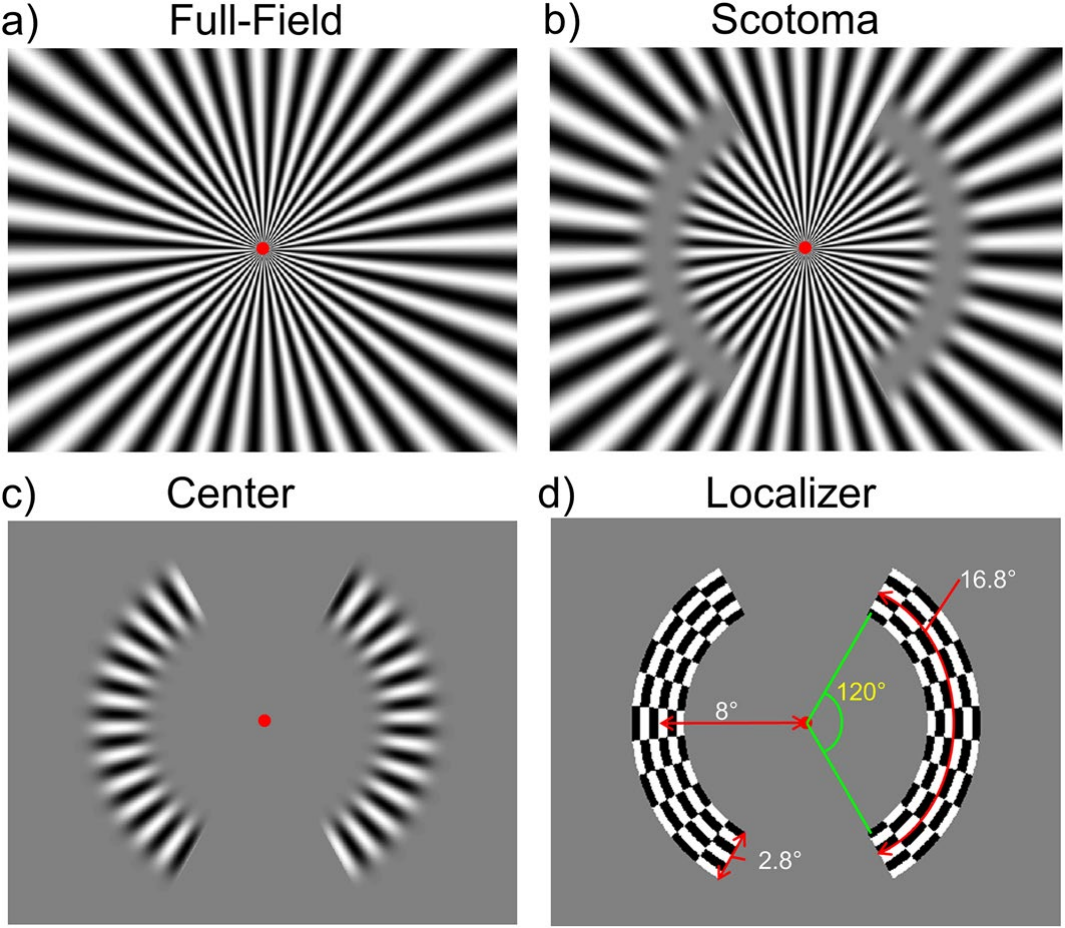
*Stimuli used in the current study*. (**a**) The full-field stimulus with radial sinusoidal grating covering both the center and surround areas. (**b**) The scotoma stimulus in which two mean-luminance blank zones (“scotomata”) located on the left and right visual fields in the 8° eccentricity periphery. (**c**) The center stimulus is the inverse of the scotoma stimulus where only the central regions corresponding to the “scotoma” contain sinusoidal grating pattern. (**d**) The high-contrast flickering (5 Hz) checkerboard pattern located in the scotoma regions used in the localizer scanning sessions. The red arrows and white numbers indicate the eccentricity (8°) and size (16.8°) in visual angle, whereas the green lines and curve and yellow number indicate the rotation angle of one of the crescents. The crescent width was set to be 2.8°. These symbols and numbers were not presented together with the stimulus during the experiment.

To further investigate the underlying neural mechanism of the lateral interaction between the center and the surround regions, in the current study, we presented three types of radial sinusoidal gratings occupying either the center, the surround, or both the center and surround of the visual fields (Fig. 1). We then measured how the brain activation varied as estimated by fMRI. We chose the fMRI technique because of its high spatial resolution. Combined with retinotopic mapping techniques, we could define voxel clusters that correspond to the first three visual areas, V1, V2 and V3 of the occipital cortex. Furthermore, using individual localizer runs, we could define the voxel clusters that correspond to the regions-of-interest (i.e., the cortical projection zone of the “center” stimuli) within early visual cortex. The effect of lateral modulation could be identified by comparing the BOLD signals for the center and the full-field gratings in the early visual cortical areas. If there was lateral excitation from the surround to the center, then the BOLD signal for the center grating should be lower than that for the full-field grating. On the other hand, if lateral inhibition predominates, then the BOLD signals for the center grating only should be greater than that for the full-field grating. Perceptual filling-in could be observed in the artificial scotomata in the surround-only gratings. Based on the previous studies, we expected to observe a change in the BOLD activation in the early visual cortex when filling-in occurred in the “scotoma” condition. We performed both univariate and multivariate pattern analyses on the data to test our hypotheses.

## Results

We presented three radial sinusoidal gratings in an event-related fMRI experiment: a Full-Field grating (Fig. 1a), a Scotoma grating with two blank crescents representing artificial scotomata on the left and right visual fields that could induce perceptual filling-in (Fig. 1b), as well as a Center grating which was the reverse of the Scotoma grating (Fig. 1c). During the stimulus presentation on each trial, one of the three gratings flickered at 5 Hz in counterphase for 10 s, followed by a 14-s blank period (see Methods for more details). The brain regions in the visual cortex corresponding to the retinotopic locations of the scotomata and center patches were identified in an independent localizer session where flickering (5 Hz) high-contrast checkerboard pattern was used (Fig. 1d). In the following sections, we analyzed the BOLD signals in these target ROIs (regions of interest). All results shown are based on brain activation within the target ROIs.

In the following sections, we first discuss the univariate analysis results, in which we compare the mean BOLD signals between the Center, Scotoma (with and without filling-in) and Full-Field conditions. Afterwards, we present the results of the multivariate pattern analysis (MVPA) conducted on the results of the scotoma trials to see if the brain activation patterns correlated with the subjective percept. Overall, participants reported seeing the scotomata being filled-in on 40–63.8% (mean 52.9%) of trials in the scotoma condition, indicating that the scotoma grating could induce perceptual filling-in.

### Univariate analysis

**Figure 2:**
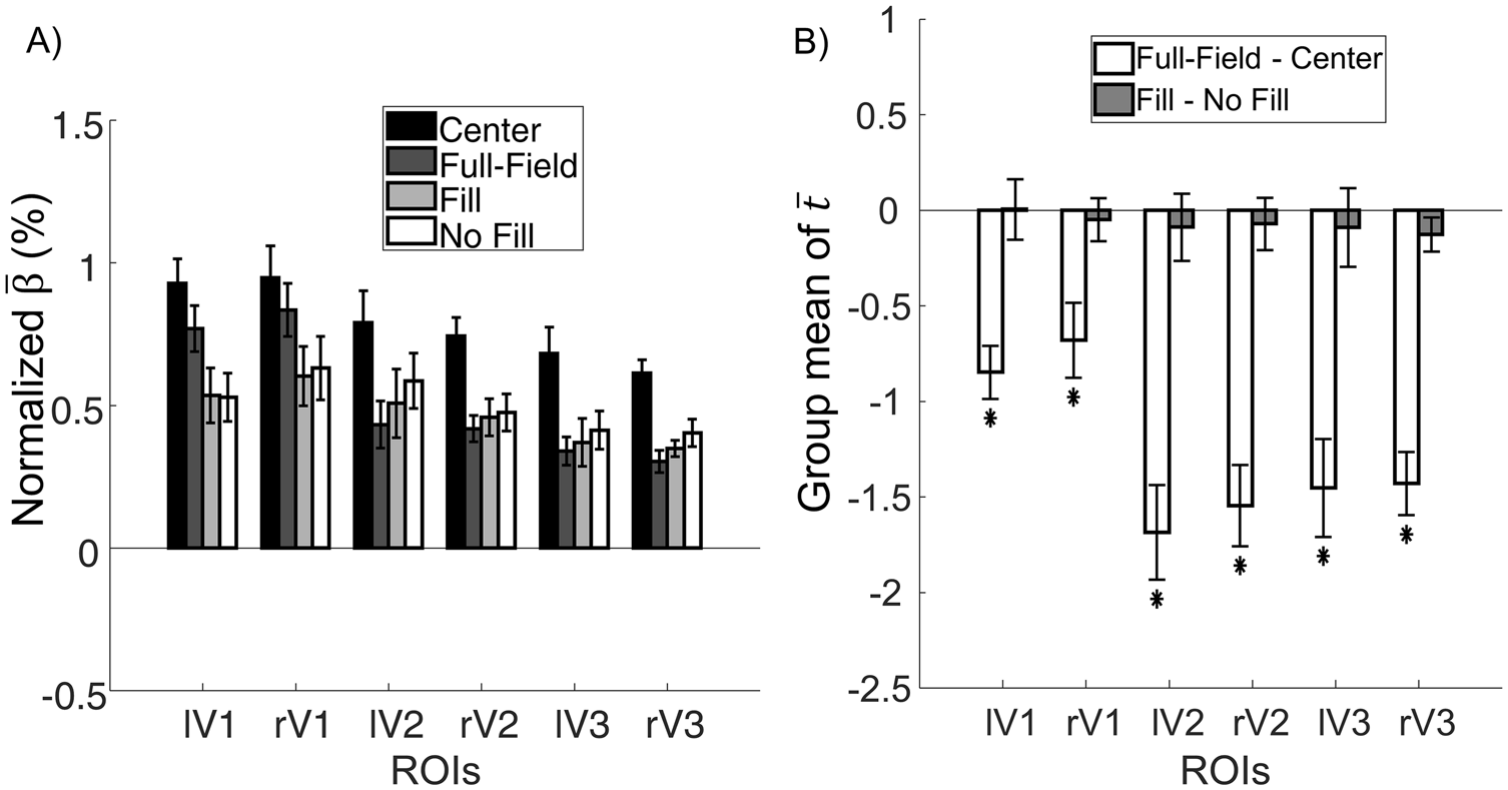
*Averaged normalized β in each condition and group mean of t-Values of the two univariate contrasts.* For visualization purpose, we normalized β for each voxel by dividing it by the baseline activation. We averaged the normalized β across voxels in each ROI at the individual-level then computed the group-level mean normalized ${\overline{\upbeta }}$ for each ROI across observers. Panel A demonstrates the normalized β of each ROI. Panel B shows the t-values of the two contrasts in each ROI. Error bar represents +/− 1 standard error. *df* = 11. *p < α = 0.0042 (after Bonferroni correction corresponding to *p* < 0.05). Results for the left (l) and right (r) visual areas V1, V2 and V3 in dorsal and ventral parts of the occipital lobe, as defined by retinotopic mapping, see Methods, are depicted.

The BOLD amplitude (β estimated by the general linear model, GLM^59^) of each condition is shown in Fig. 2A. We focus on the results of two condition contrasts (Fig. 2B). First, to examine the effect of adding a surround to the center grating patches on the BOLD activation, we compared the activation levels of the target six ROIs (corresponding to the projection zones of the “center” or “scotoma” regions) between the Center and Full-Field conditions (see Fig. 1). As reported earlier^16^, we expected to find a reduced BOLD signal in the ROI regions in the Full-Field condition compared to the Center condition. Second, we examined whether the amplitude of the BOLD response was different between trials with filling-in report and those without. The group mean t-values of both contrasts are plotted in Fig. 2B for the left (l) and right (r) ROIs in retinotopically defined V1, V2 and V3 regions of the occipital lobe.

We performed one-tail t-tests on the group mean t-values compare to zero for each contrast for all ROIs. In the case of Full Field-Center contrast, the mean t-values were significantly below zero for all ROIs (lV1: *t*(11) =  − 6.13, *p* < 0.001, Cohen’s *d* = 1.77; rV1: *t*(11) = − 3.47, *p* = 0.003, Cohen’s *d* = 1.00; lV2: *t*(11) =  − 6.80, *p* < 0.001, Cohen’s *d* = 1.96; rV2: *t*(11) = − 7.26, *p* < 0.001, Cohen’s *d* = 2.10; lV3: *t*(11) =  − 5.67, Cohen’s *d* = 1.64, *p* < 0.001; rV3: *t*(11) = − 8.63, *p* < 0.001, Cohen’s *d* = 2.49), implying that the activation in the full-field condition was weaker than in the center condition, suggesting an inhibitory lateral modulation effect. In the “scotoma” condition, the Fill–No-Fill univariate contrast did not reveal any significant *t*-test results (lV1: *t*(11) = 0.03, *p* = 0.49; rV1: *t*(11) =  − 0.43, *p* = 0.34; lV2: *t*(11) =  − 0.50, *p* = 0.31; rV2: *t*(11) =  − 0.52, *p* = 0.31; lV3: *t*(11) =  − 0.43, *p* = 0.34; rV3: *t*(11) =  − 1.4, *p* = 0.09), suggesting that the overall activation level did not depend on the subjective percept of the scotoma gratings.

**Figure 3:**
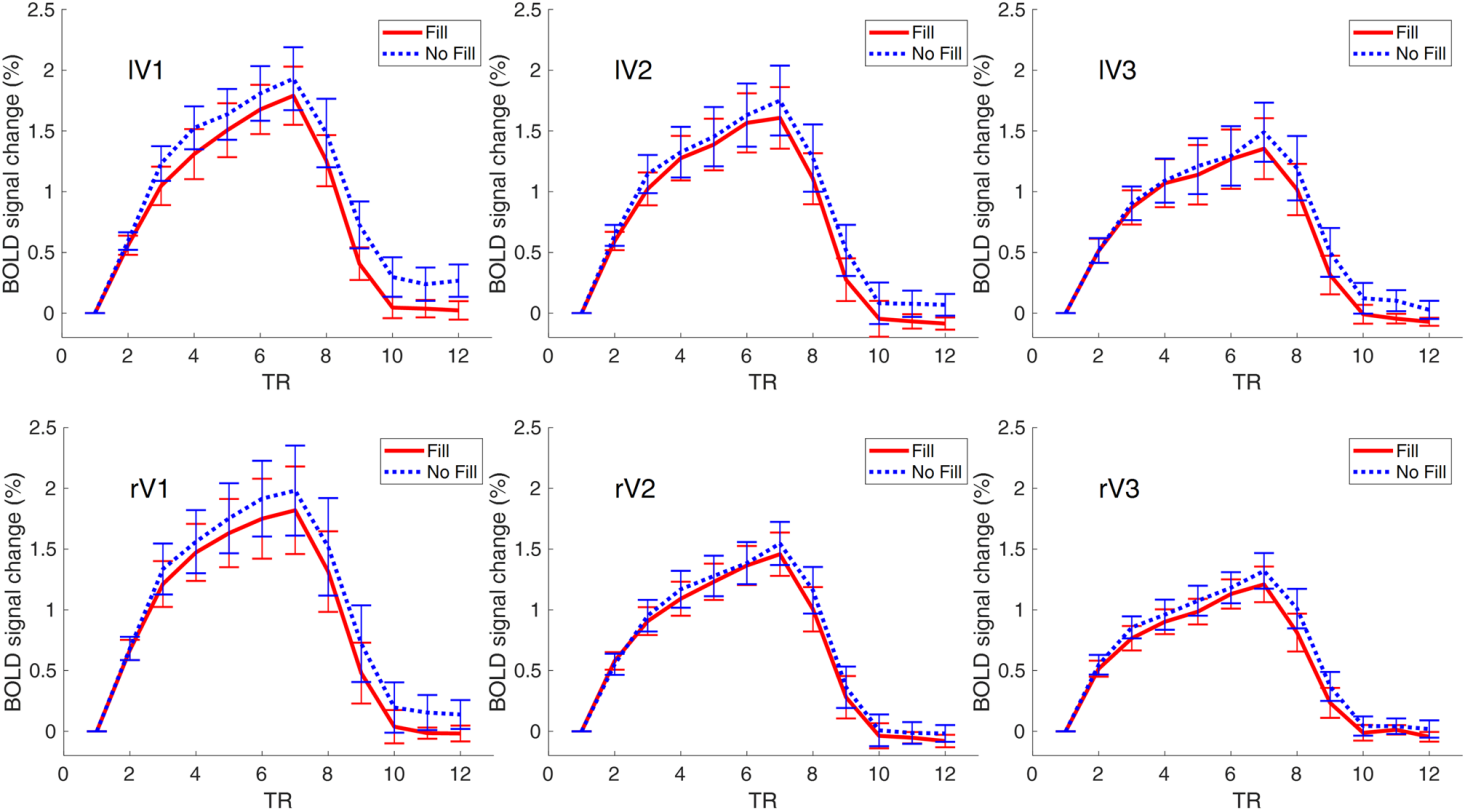
*Time course of filling-in and no filling-in conditions.* We plotted the percentage of BOLD signal change relative to the first TR across the 12 time points in each ROI. We first calculated the signal change of BOLD response relative to the first TR relative to the stimulus onset for each trial in each condition and averaged them to get the individual time course of percentage BOLD signal change. At the group-level, we averaged these time courses of all subjects to get the group mean time course. The error bars represent the +/− standard error across participants. Results for left (l) and right (r) visual areas V1, V2 and V3 are shown in the upper and lower panels, respectively.

To examine whether there was a difference in the time course Fill and No Fill conditions, we extracted the BOLD signal across the 12 TRs (5 TRs/10 s with stimulus on, 7 TRs/14 s with stimulus off) in the filling-in and no filling-in trials of each ROI for each observer and plotted them in the Fig. 3. It can be seen that the time courses of the two conditions were quite similar for all ROIs.

**Figure 4:**
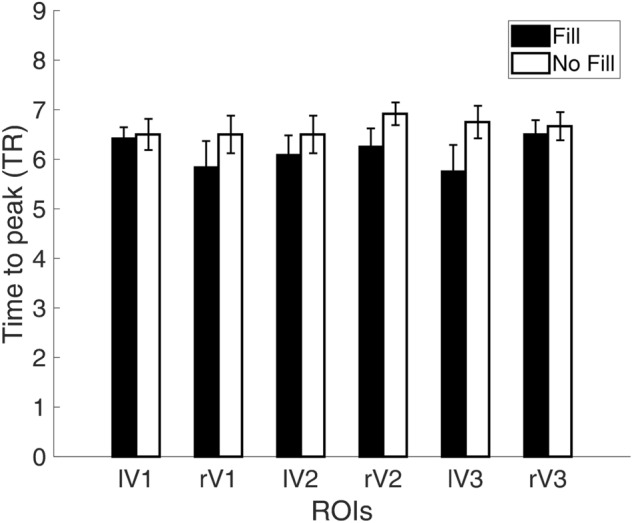
*Averaged time to peak for Fill and No Fill conditions.* We extracted the time to peak (the TR with the peak activation after stimulus onset) for each trial in each ROI then calculated the averaged time to peak for each observer in the Fill and No Fill conditions. The figure shows the average time to peak in TR across observers in each ROI. The error bars represent the +/− standard error across participants.

In addition, we plotted the mean time to peak of the Fill and No Fill conditions in Fig. 4. We calculated the difference of averaged time to peak between the Fill and No Fill conditions and performed two-tailed paired t-tests for each ROI. None of the p-values were lower than the α value 0.0042 after Bonferroni correction corresponding to *p* < 0.05 (lV1: *t*(11) =  − 0.43, *p* = 0.67; rV1: *t*(11) =  − 1.54, *p* = 0.15; lV2: *t*(11) =  − 1.16, *p* = 0.27; rV2: *t*(11) =  − 2.35, *p* = 0.04; lV3: *t*(11) =  − 1.91, *p* = 0.08; rV3: *t*(11) =  − 0.80, *p* = 0.44), suggesting that there was no difference in time to peak between the filling-in and no filling-in trials.

### Multivariate analysis

**Figure 5:**
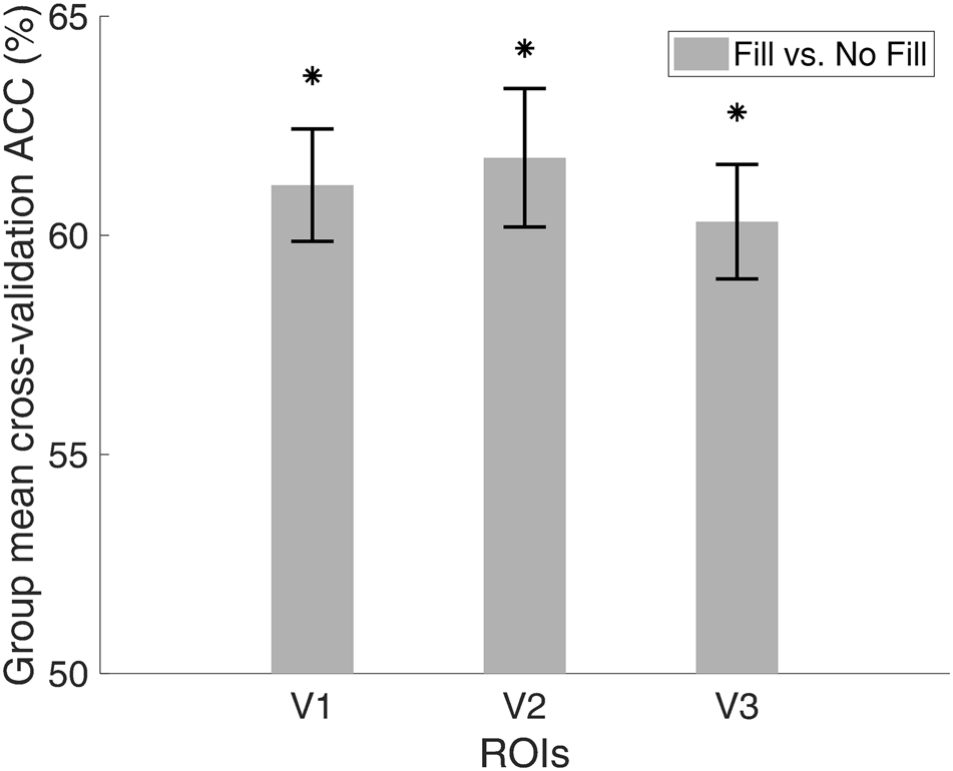
*The group mean of cross-validation accuracy of MVPA.* The figure presents the group mean of the cross-validation accuracy. Error bar represents +/− 1 standard error. *p < α = 0.017 (after Bonferroni correction). Results for the visual areas V1, V2 and V3 are depicted (see Methods for details of ROI definition).

It is possible that differences in filling-in percept is better reflected by the activation patterns across all voxels within the projection zone of the “scotoma” region. To test this hypothesis, we performed a multivariate pattern analysis by training an SVM linear classifier to differentiate between the Fill and No-Fill trials of each participant. We then averaged the cross-validation accuracy of all twelve participants and tested this group mean classification accuracy against a null distribution generated by permutation. Figure 5 shows the group mean of the cross-validation averaged accuracy. The classification accuracy was considered to be significant if the averaged accuracy corresponded to a *p* value below an α value of 0.017 (after Bonferroni correction corresponding to *p* < 0.05) in the null permutation distribution.

Our results showed that for all targeted ROIs, the model can be successfully trained to differentiate between filling-in and no-filling-in trials (all *p* values were below 0.001), indicating that the BOLD activation pattern in the two conditions carry enough information for the SVM models to predict the presence or absence of filling-in with more than 60% accuracy.

### Fixation stability test

To make sure that the differences of BOLD signal levels or activation patterns found between conditions did not originate from variation in how well the participants fixated during the stimulus presentation, we recorded the eye movement of the observers. Two observers, P1 and P2, performed the same experiment outside of the scanner after the main experiment while their eye movements were recorded by an eye tracker. The eye positions of the other four participants, P3 to P6, were recorded during the fMRI main experiment (see “Methods” for more details). P3 to P6 were not available for the out-of-scanner eye tracking control experiment. To preprocess the raw eye tracking data recorded during stimulus presentation, we removed eye positions that indicate missing data, blinks, or gaze positions beyond the stimulus screen to remove artifacts. We also ruled out eye positions that indicate eye movement between two stimulus frames that exceeded eye velocities of 30° per second to exclude large saccades^60^. We calculated the bivariate contour ellipse area (BCEA) values to evaluate the level of fixation stability^60–63^ in our different stimulus conditions. The BCEA is defined by,

1 $$BCEA = 2k\pi \sigma_{H} \sigma_{V} \left( {1 - \rho^{2} } \right)^{0.5}$$

in which $k$ is a constant determining the probability area as in the next equation:

2 $$P = 1 - e^{ - k} ,$$

with $e$ the base of the natural logarithm and $P$ the probability area. $\sigma_{H}$ and $\sigma_{V}$ are the standard deviations of the horizontal and vertical eye positions recorded, whereas $\rho$ is the Pearson’s correlation coefficient between the eye positions in the two directions. We used $k = 1$, where the BCEA encompasses about 63.2% of the fixations, to estimate the BCEA value.

**Figure 6:**
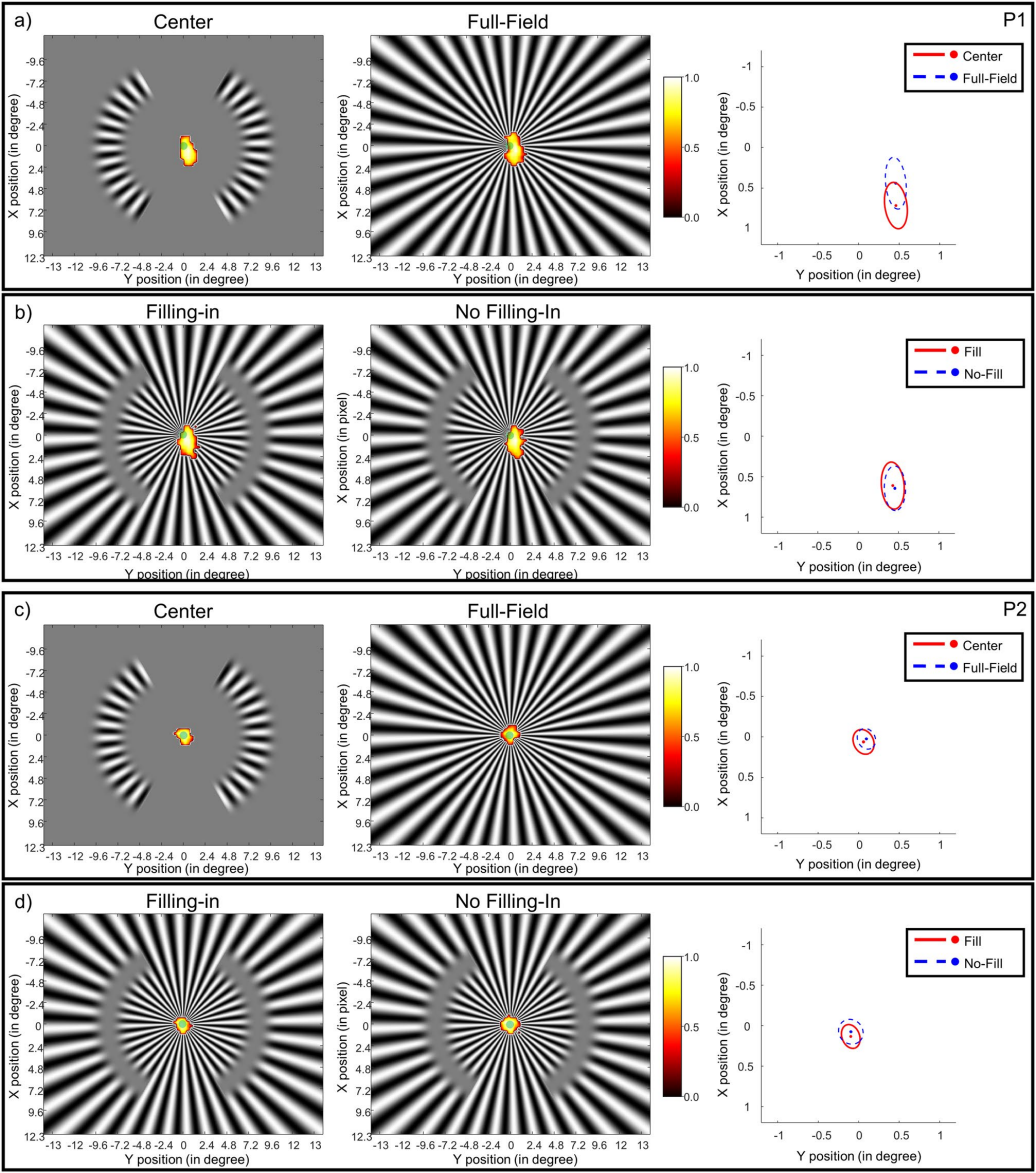
*Fixation data of participant P1 and P2 recorded in the out-of-scanner control experiment*. Panel (**a**) and (**b**) demonstrates the data of P1, whereas panel (**c**) and (**d**) the data of P2. Panel (**a**) and (**c**) show the data of Center and Full-Field conditions. The left and middle plots in panel (**a**) and (**c**) represent the density maps of the Center and Full-Field gratings. The density value is shown according to the color bar on the right side, with white corresponding to 1.0 and black 0.0. The green dots in the middle of the gratings represent the fixation point. The right plots in (**a**) and (**c**) shows the BCEA ellipses of the Center, with the red solid-line oval and the red dot, and the Full-Field condition, with the blue dashed-line oval and the blue dot. Panel (**b**) and (**d**) show the data of Fill and No-Fill conditions, with the left plots presenting the density map of the Fill and the middle the No-Fill condition. The right plots in (**b**) and (**d**) shows the BCEA ellipses of the Fill, with the red solid-line oval and the red dot, and the No-Fill condition, with the blue dashed-line oval and the blue dot.

We estimated the BCEA value for each participant in each stimulus condition. We compared the BCEA values between the center and full-field conditions, the Center–Full-field contrast, as well as the contrast in the filling-in and no-filling-in trials. We performed two one-way repeated measurement ANOVA on the two contrasts and found no significant difference between the BCEA values in different conditions (Center–Full-Field: *F*(1, 5) = 1.6, *p* = 0.26; Fill–No Fill: *F*(1, 5) = 0.39, *p* = 0.56). Due to the small sample size ($n = 6$), besides *F*-tests, we used random sampling with replacement, i.e. bootstrapping method, to generate a null distribution out of 10,000 iterations. We calculated the *p* values of the two mean differences of the two contrasts (i.e., center vs. full-field and filling-in vs. no filling-in) in their corresponding distributions and compared them to an α value of 0.025 (two-tail). The tests showed that both *p* values were not significant (Center–Full-Field: mean = 1.84, *p* = 0.40; Fill–No-Fill: mean =  − 0.49, *p* = 0.48). Both parametric and non-parametric statistics results indicate that there were no significant differences in the fixation stability in the different stimulus conditions. After establishing that no difference was found in the fixation variability, we further examined if the positions of the BCEA ellipses’ center differed between conditions in the two contrasts. We found that for both contrasts, in all six participants, the BCEA ellipse center of one condition fell within the contour of the other condition, suggesting that the fixational stability was more or less constant across conditions. The fixation density map and the BCEA ellipses of two representative participants are presented in Fig. 6. The details of how we constructed the density map and the summary of the BCEA sizes and positions of different stimulus conditions of all six participants can be found in the Supplementary File (File S1). Our fixation stability test results indicated that the difference in BOLD activation we observed in the Center and Full-Field conditions as well as between the Fill and No-Fill trials in the “scotoma” condition was unlikely to be explained by variability in fixation stability.

## Discussion

To better understand the cortical response associated with the lateral modulation effects such as lateral inhibition and filling-in phenomenon, we presented to participants radial sinusoidal gratings during fMRI. Three types of gratings were used (Fig. 1): a Center grating with two radial sinusoidal grating patches centered at 8° eccentricity, one in each visual hemifield, a Scotoma grating that was the reverse of the Center grating with two blank “scotoma” regions embedded in the radial gratings, and a Full-Field grating with a radial grating extending over the central and near peripheral visual field.

Comparing between the Center and Full-Field grating allowed us to observe the lateral modulation effect when the surround pattern was added to the center. In a univariate analysis on the BOLD responses in the voxel clusters corresponding to the “center” or scotoma regions of the display we found evidence for lateral inhibition in V1 to V3 regions (Fig. 2). This lateral inhibition effect supports our previous psychophysical experiments^16,17^, where we discovered that adding a surround to the adapter center decreased the adaptation effect, suggesting the existence of surround suppression. Similar lateral inhibition has been reported in other neuroimaging studies^48,54^.

In the “scotoma” conditions, participants were requested to report whether they experienced “filling-in”, i.e., the percept of a continuous radial sinusoidal grating, allowing us to compare the BOLD activation between the trials when filling-in was perceived and when it was not. Contrary to previous studies^56,57^, we did not find a significant BOLD amplitude difference between trials with and without filling-in. Unlike the homogeneous disk used in Mendola et al.^57^ or the random flickering noise in Weil et al.^56^, we used blank crescents positioned left and right of central fixation. Therefore, the filling-in percept perceived by our participants corresponds more to a “fading” of the blank scotoma region instead of a complete disappearance of homogenously illuminated region. Moreover, some participants reported experiencing partial filling-in in the scotoma regions or that different parts of the scotoma appeared to be filled-in at different time points. Our forced-choice task did not allow for the detection of such nuisances in the filling-in percepts. It is also possible that we had higher level of noise in the current experiment that the filling-in effect was not discernable from the noise. We also did not observe a difference in the time course or the time to peak in the Fill and No Fill conditions (Figs. 3, 4). In the multivariate analysis, however, the linear SVM classifier trained on the filling-in and no-filling-in trials did successfully predict the subjective percept in the test trials in V1, V2, as well as V3 with more than 60% accuracy level (Fig. 5). Such results suggest that even though the mean BOLD signals did not differ between the two conditions, differences in brain activation patterns were present in early visual cortex.

To rule out the alternative explanation that our findings related to variation in fixation stability across our stimulus conditions, we recorded the eye positions of six out of the twelve participants during the different stimulus conditions. An analysis of fixation stability across conditions indicated that it did not differ for the center and full-field conditions or for the filling-in and no-filling-in trials in the “scotoma” condition, indicating that the effects we found in BOLD activations were unlikely to arise from differences in fixation stability.

One limitation of the current paradigm is that we did not monitor the attention state of the participants on the trial-by-trial base. Since a scotoma trial was classified as a filling-in or a no filling-in trial depending on the response of the participants, it could be misclassified if the participants failed to respond due to a momentary lapse of attention rather than a lack of filling-in percept. The lack of attention would then lead to a lower filling-in report rate than expected. Having said that, any effect of attention does not seem to have been substantial in our experiment. We found that the filling-in rate remained around 50%, close to what we expected estimated from the pre-tests, for all experimental runs, suggesting that there was not much fluctuation of the filling-in reporting rate across runs. This could serve as indirect evidence that the loss of attention did not contribute systematically to the percepts reported on the no filling-in trials. In addition, in the Center and Full-Field conditions, participants were not asked to make a filling-in judgment. Consequently, the allocation of attention in these two conditions might be different than that evident in the scotoma condition.

To conclude, we used a simple paradigm to study the lateral modulation effect in vision. We found evidence of lateral inhibition in V1 to V3 regions when a surround pattern was added to the center grating. We also discovered that the brain activation pattern was different when filling-in was perceived from when it was not perceived. During the main experiment, all our stimuli had a constant contrast level. It is well known that the magnitude and direction of the lateral modulation effect can vary with the contrast and orientation of the center/surround stimuli^8,15,17,19,21,54,64^. Future studies might incorporate such variations to determine their effect on the resultant BOLD response in early visual cortex. Other related phenomena like contour erasure^65,66^ or water color illusion^35,67,68^ could exhibit neural correlates similar to those reported for filling-in.

## Methods

### Participants

Twelve participants including one author, YSL, coded with P1, and eleven naïve observers unaware of the experiment purpose, aged between 20 and 31, took part in the fMRI experiment. All of them had normal or corrected-to-normal vision. Every participant completed three sessions: the ROI localizer session, the pre-test session, and the main experiment session. P1 and P2 participated in an additional eye tracking control experiment outside of the scanner. Informed consent was received from all observers before MRI scanning and monetary compensation was provided after each session as rewards for the naïve participants. The experiment was approved by the IRB of the National Taiwan University Hospital and was performed according to the Declaration of Helsinki on human experimentation.

### Apparatus and image acquisition

During the MRI scans, all stimuli and experiment procedure were presented on a pair of MR-compatible head-mounted goggles (Resonance Technology, USA), delivered by a Windows 7 PC in the MR control room. Each of the two goggle displays (one for each eye) had a resolution was 800 × 600 with one pixel size set to 0.035° visual angle and a refresh rate of 60 Hz. The visual acuity of the observer could be corrected with the convex lenses inserted in front of the goggle displays. Two MR-compatible response boxes were placed below the left and right hands of the participants to record their behavioral responses with respect to the presence or absence of filling-in.

The T2* weighted images were collected with the Siemens Prisma 3T scanner in National Taiwan University with a 64 channel surface head coil. The anatomical images (T1-weighted, MPRAGE sequence, TR = 2000 ms, TE = 2.3 ms, FA (flip angle) = 8°, image matrix = 256 × 256 × 192, FOV = 240 × 240, voxel size = 0.93 × 0.93 × 0.93 mm) were acquired at the beginning of each scan to construct the anatomy brain model of each participant. The functional images (T2*-weighted) were collected in 32 transverse planes aligned with the AC-PC (anterior commissure-posterior commissure) direction. An echo-planar imaging sequence^69^ was used to acquire 32 transverse planes parallel to the AC-PC (anterior commissure-posterior commissure) for the functional data (TR = 2000 ms for the pretest and the main experiment, TR = 3000 ms for the retinotopic and scotoma localizer scans, TE = 25 ms, FA = 90°, image matrix = 96 × 96 × 32, FOV = 192 × 192, voxel size = 2 × 2 × 2 mm). An extra low-resolution T1 inplane (TR = 595 ms, TE = 6 ms, FA = 70°, image matrix = 192 × 192 × 32, FOV = 192 × 192, voxel size = 1 × 1 × 2 mm) acquired in planes identical to the functional images was collected to facilitate alignment between the functional and anatomical images.

### Eye tracking apparatus

To record the participants’ eye positions during stimulus presentation, we used two eye trackers in the current study. The first is the MR-safe ViewPoint EyeTracker system by Arrington Research^®^, Inc (www.ArringtonResearch.com) with 60 Hz data acquisition rate controlled by a Windows 7 PC which recorded eye positions during the stimulus presentation in the scanner. The second is the EyeLink 1000 (SR Research Ltd., Canada) system used in an out-of-the scanner control experiment controlled by a Windows 10 PC. The data acquisition rate was 1000 Hz. The stimuli for the out-of-scanner control measures were presented on a 17-inch EIZO FlexScan S1910 monitor calibrated with a PR-655 SpectraScan^®^ photometer, with a refresh rate of 60 Hz and a resolution of 1280 × 1024 with one pixel size of 0.023° visual angle. The fixation stability test conducted outside of the scanner was carried out in a dimly lit room.

### Stimuli

To maximize brain response induced by the visual stimuli, instead of using small sinusoidal patterns presented in one visual quadrant like was done in our previous psychophysics experiments^16,17^, we generated radial sinusoidal gratings (patterns expanding from the fixation center to the display border) that occupied larger regions of the visual field. Three such radial sinusoidal gratings were used in the experiments: a Full-field grating (Fig. 1a), a Scotoma grating (Fig. 1b), and a Center grating (Fig. 1c). The Full-Field radial grating were defined by the following equation,

3 $$G = B + BC\cos \left( {2\pi f\theta } \right),$$

where $\theta$ is the angular coordinate in the polar coordinate system, $B$ the mean luminance, $C$ the stimulus contrast, and $f$ the spatial frequency. In the current study, $f$ was set to 0.1 cycle-per-degree. Note that the degree in the unit refers to the rotation angle, not visual angle. Therefore, there were 36 cycles across the 360° radial grating pattern. The Center grating was created by multiplying the grating in Eq. (3) with the following window:

4 $$W = e^{{ - \left( {\frac{{\left( {r - r_{E} } \right)^{2} }}{{2\sigma_{r}^{2} }}} \right)^{P} }} ,$$

in which $r$ is the radial coordinate of the polar coordinate system, $r_{E}$ determines the eccentricity of the center of the window and $\sigma_{r}$, the radial scaling parameter, decides the width of the window. In the current study, the $r_{E}$ was set to 8° and $\sigma_{r}$ 1°. We masked out the grating pattern positioned between − 30° and + 30° rotation angle from the vertical meridian to separate the center grating into two crescent patches that span across 120° rotation angle (16.8° length in visual angle), one on each side of the visual field. The scotoma grating was the reversed pattern of the center grating, where two scotomata were positioned at the patches’ locations.

The power of the Gaussian mask, $P$ value in Eq. (2), determines the sharpness the edges of the patches in the center grating and the scotomata in the scotoma grating. In a pilot study, we tested scotoma stimuli of different $P$ values on two observers (P1 and P2) and found that when $P$ was equal to 3, perceptual filling-in was perceived about 50% of the time for − 6 dB (50%) surround luminance contrast level. Thus, we chose such $P$ value for the stimuli used in the formal experiments. The localizer pattern (Fig. 1d) was created by applying the same window in Eq. (2) but with a power value of 1000 to create a sharp edge in the radial direction. The width of the scotoma/center patches were 2.8°, wide enough to cover the receptive fields of early visual areas that are about 0.5° in V1, 1.9° in V2, and 3.4° in V3 at 8° eccentricity according to the estimates from single-neuron recording studies in primate visual cortex (meta-analysis shown in Fig. 9 in Smith et al.^70^, based on the data of^71–76^).

### Procedure

#### Luminance contrast calibration task in the scanner

During a calibration session of the MRI-safe goggles, the left side of the screen was filled with a homogeneous square whereas the right side a high spatial frequency flickering square wave grating of maximal contrast. The observers were to adjust the luminance level of the homogeneous square to match the perceived luminance of the grating. We then fitted a power function between the subjective luminance percept and the display values to determine the gamma value used for calibrating the visual display during the experiment, i.e. gamma correction. The deduced gamma value was then used to compute the linear look-up table for the goggles for the subsequent experiment sessions. Such calibration task was conducted at the beginning of each scanning session.

#### ROI localization

The regions of interest (ROIs) in the visual cortices of each observer were identified before the experiment sessions with the following method.

1. *Retinotopic mapping* To identify the first-tier visual areas (V1, V2 and V3), we combined a rotating wedge and an expanding ring made of high-contrast checkerboards patterns that moved across the visual field^77,78^. The patterns flickering in counterphase at 4 Hz. The rotating wedge spanned across 45° and rotated 22.5° in clockwise direction every TR (3000 ms), thus mapping the whole visual field in every 48 s, whereas the expanding ring of a width of 1.77° moved from the foveal area (0.25°) to the periphery (10.6°) with a step of 0.86° every TR, requiring 36 s to finish the whole cycle. One retinotopic mapping run took 288 s (96 TRs) to complete, resulting in 6 repetition cycles for the rotating wedge and 8 cycles for the expanding ring. Each run was repeated for at least four times for each participant.
2. *Scotoma localizer* To target the brain regions corresponding to the scotomata of our stimuli, we conducted an independent localizer scan in which two high-contrast checkerboard crescent patterns flickering at 5 Hz located at the scotoma regions (Fig. 1D) were presented in an on-and-off manner with on and off period taking 6 TRs (TR = 3000 ms) each and repeating six times resulting in a total of 72 TRs per scan. The scotoma scan was repeated at least two times for each participant.

To make sure the observer maintained steady fixation during each scan, we added a center fixation task during the stimulus presentation in the localizer runs. Participants were to count the times the fixation dot changed color during the scan and reported the number at the end of each scan. Only runs with high accuracy (over 95% correct) fixation task performance on the central counting task were retained and analyzed. The same performance criterion was applied in the subsequent pre-test and main experiment scans to ensure steady fixation of participants.

#### Pre-test

The purpose of the pre-test session was to adjust the luminance contrast level for each observer until they could perceive filling-in about 50% of the time for the Scotoma condition (Fig. 1b). Thus, only the scotoma stimulus was used in the pre-test scans. Each run contained 10 trials; on each trial, the surround grating flickered at 5 Hz for 10 s and then followed by a 14-s blank period. The participants were instructed to press a button on a response box held by their left-hand whenever they perceive a filling-in during the scotoma trials. The scotoma trials with the filling-in button pressed were categorized as trials with perceived filling-in, whereas the trials without the filling-in button pressed were categorized as no filling-in trials. In addition, they were to press another button on response box at their right hand whenever the fixation color changed from green to red during the whole experiment. If the participants reported perceiving filling-in more than six times out of the ten trials, the inducer contrast would increase in the next run. Otherwise, if, they should perceive filling-in less than four times out of ten, the inducer contrast would decrease on the next run. Each observer participated in at least four runs. We then used the most suitable luminance contrast for all three types of radial gratings for each participant in the main experiment. As a result, all stimuli in the main experiment were of the same luminance contrast. The pretest was complete in 20 to 30 min.

#### Main experiment

In the main experiment, all three radial sinusoidal gratings were used. There were 20 trials in each run, containing five center grating trials, five full-field grating trials, and ten scotoma grating trials in random sequence. On each trial, one of the three stimulus gratings flickered at 5 Hz for 10 s (5 TRs) followed by a 14-s (7 TRs) blank period. A fixation task, in which the participants were to press a button with their right hand when the fixation changed color, was performed during the whole experiment to control the attention of the participants. All participants had an accuracy for this task greater than 95%. In addition, in scotoma trials, participants were to press a button on their left hand when filling-in was perceived during the scotoma grating presentation. Participants were instructed to keep their eyes open during the stimulus presentation and blink only after the stimulus disappeared during each trial throughout the experiment. Each participant completed eight runs in the main experiment, resulting in forty center and full-field trials, and eighty scotoma trials that contained trials with filling-in percept and those without. The main experiment took around seventy minutes to complete.

#### Fixation stability test

To test whether there were differences in eye movements and fixation stability during different conditions, we collected eye-tracking data in four participants (P3 to P6) during MRI scanning. Calibration of the eye tracker was performed at the beginning of the fMRI main experiment. During the calibration process, the participants were instructed to fixate a white calibration dot target positioned on a mean luminance background that appeared in one of the sixteen positions in a random sequence. The calibration dot changed to the next position once it recorded stable eye fixation. The fMRI main experiment was performed after good calibration was achieved. In the remaining two participants (P1 and P2), we conducted an additional control experiment with the same stimulus conditions outside of the scanner. The experiment procedure was the same as the main experiment except that calibration and validation sessions were performed before each run. We performed a nine-dot calibration procedure, where participants were to fixate on the white calibration dot target in a mean luminance background that appeared in one of the nine calibration positions in random order. The target moved to the next position only when it recorded stable fixation. After the calibration was accepted, a validation procedure where participants were to fixate the target dot sequentially presented in the same nine positions was performed again. The eye tracker system then computed the deviations between the target positions and the computed fixation positions according to the calibration estimates. The calibration/validation cycle was accepted only when none of the deviations of the nine positions exceeded 0.5 degree. The control experiment was performed after good calibration/validation results were achieved. Eye positions were recorded during the 10-s ON period on each trial during the experiment.

All stimuli generation and experiment procedure controls were done with MATLAB (MathWorks, Inc., Natick, MA) with PsychToolbox Version 3 (http://psychtoolbox.org/).

### Data analysis

For the anatomical images, we reconstructed cortical surface models for all participants with Freesurfer^79–81^, which involved automated reconstruction of each MPRAGE anatomical scan that separated the brain into gray- and white-matter surfaces. As for the functional imaging scans, after DICOM conversion done by the MRIcron (https://www.nitrc.org/projects/mricron), preprocessing steps that include motion correction referenced to the first image and slice time correction were done with SPM8 (http://www.fil.ion.ucl.ac.uk/spm/software/spm8/), a MATLAB-based toolbox. No spatial normalization or spatial smoothing was performed for the individual brain images for univariate and multivariate analyses. The analyzed images were then fed to the mrVista toolbox (https://web.stanford.edu/group/vista/cgi-bin/wiki/index.php/MrVista) where co-registration and data visualization were performed. The general linear model (GLM) and further statistical analyses were done by customized MATLAB codes according to the standard GLM methods^59^. To test the target contrasts between conditions, we calculated the t-values based on the mean and variance of each contrast (difference in β values between the two conditions) for each voxel. We averaged the t-values across all voxels for each ROI as one datapoint for each observer. We then performed a *t*-test on these averaged t-values at the group-level for each ROI (Fig. 2B).

**Figure 7:**
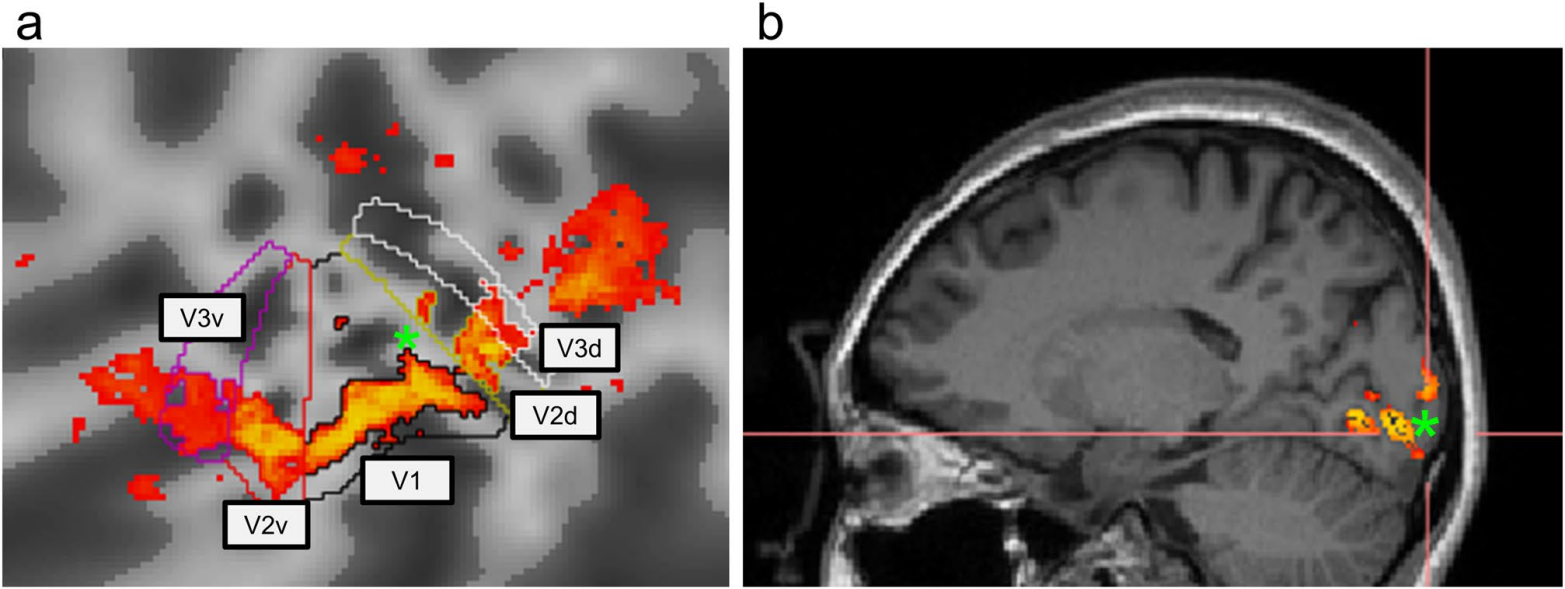
*Demonstration of ROIs of the left hemisphere of one participant.* This figure shows the examples of defined ROIs of the left hemisphere used for data extraction and analysis in the current study. Panel a represents the ROIs in an inflated and flattened view. The color outlines indicate the contours of each early visual area delineated from the retinotopic mapping data. The orange patches demonstrate the regions activated by the “scotoma” localizer. Panel b shows the same activation pattern in the sagittal view of the anatomical brain. The green asterisks correspond to the same position in the brain on the two different views.

We used the mrVista toolox to delineate early visual areas for each participant based on the images of the retinotopic mapping localizer sessions. We used the phase-reversals according to the rotating wedge data to define the boundaries between visual areas. For the first-tier visual area, we defined five visual areas: V1, dorsal V2, ventral V2, dorsal V3, and ventral V3 for each hemisphere. Then we restricted the area responding more to the scotoma localizer than the gray background within each retinotopically defined ROI. A voxel was considered activated if the t-statistics of the regression coefficient exceeded 5.09 ($\left| r \right| > 0.52$). The criterion corresponded to a one-tail Bonferroni corrected α-level at 0.05 for each voxel considering the total number of gray matter voxels. Figure 7 demonstrates such restricted ROIs in the left hemisphere of one participant. To make sure that the voxels in these ROIs received minimal contribution from the surround region outside of the scotoma localizer center, we further restricted the ROI by leaving only the voxels that had greater activation to the Center grating (Fig. 1c) than to the Scotoma grating (Fig. 1b) used in the main experiment. We achieved this by computing the activation difference between the Center grating and Scotoma grating and removing the voxels with activation in the Scotoma condition greater than that in the Center condition. We combined the ventral and dorsal subregions of V2 and V3 together as one V2 and V3 areas for data analysis, resulting in three ROIs per hemisphere and six in total for each participant for univariate analysis (Figs. 2, 3, 4). For the multivariate analysis, to ensure we had enough voxels left in each ROI as features for SVM model training, we further combined corresponding ROIs in both hemispheres together resulting in three ROIs, V1, V2, and V3 regions. Therefore, the MVPA results were discussed for only three ROIs (Fig. 5).

To examine if there was a difference in the activation pattern, we used LIBSVM toolbox^82^ to perform a multivariate pattern analysis (MVPA) on the trial-by-trial data^83^. We first transformed the unsmoothed preprocessed data of all voxels in each ROI into z-scores by normalizing the data across the time points in each run. We then rescaled the z-scores to range 0–1 and averaged the BOLD activation from 3rd to 7th TRs relative to stimulus onset for model training and testing. The choice of such activation period was to target the peak BOLD response. For each participant, we used leave-one-out method to train a linear SVM classifier on the data of filling-in and no-filling-in trials of seven runs, which included 70 trials, then tested the model on the remaining run, which contained 10 trials. We performed such training and testing procedure eight times until all runs were being tested once and then we averaged the classification accuracies across the eight cross-validations. To examine the classification result, we performed a permutation test^84–86^. We randomly relabeled the test and train data in each ROI of each participant with respect to “filling-in” and “no filling-in” trials for every cross-validation and average the prediction accuracy across the eight repetitions. We then computed the group mean value of the averaged cross-validation accuracy across all participants. Such a process with random assignment was repeated 5000 times, leading to a distribution of group mean accuracy for each ROI.

## Supplementary Information

Supplementary Information.

## Data Availability

The datasets generated and analyzed in the current study are available from the corresponding author upon reasonable request.
