# Supplementary material for: Neural correlates of lateral modulation and perceptual filling-in in center-surround radial sinusoidal gratings: an fMRI study
Source: Sci Rep. 2022 Sep 27;12:16143. doi: 10.1038/s41598-022-20592-y (PMC9515077; doi:10.1038/s41598-022-20592-y)
Supplement: Supplementary file 1 — Supplementary Information. [file 41598_2022_20592_MOESM1_ESM.docx]

Supplementary

We summarize the BCEA data of each stimulus condition, including the sizes and positions, in Table S1, of the six participants who we recorded eye tracking data from. In Figure 6 of the main manuscript, we show the fixation density maps as well as the BCEA ellipses of the four conditions.

The fixation density maps shown in Figure 6 in the main manuscript were created by dividing the whole stimulus screen into numerous cells. For the out-scanner control experiment where the resolution was $1280\times1024$, each cell was $16\times16$ square pixel large, resulting in 80 cells on the x-axis and 64 cells on the y-axis. For the in-scanner eye tracking recording where the resolution was $800\times600$, each cell was $10\times10$ square pixel, leading to 80 cells on the x-axis and 60 on the y-axis. We then counted the frequency of eye position located in each cell and divided each cell frequency by the highest frequency of all cells to calculate the eye position density. We overlayed such fixation density map on top of the stimulus.

As for the BCEA ellipses, we performed principle component analysis to recover the BCEA ellipses of all conditions (Niehorster et al., 2020). When $k$ in the equation (3) in the main manuscript is equal to 1, the ellipse covers 63.2% of the fixation points, and the eigenvalues in the major and minor eigenvectors represent the squared lengths of the two axes. We plotted the ellipses of different conditions on the right plot in each panel in the following figures.

In our data, although there was some individual difference in terms of the density maps and BCEA ellipses, within each participant the fixation distribution and positions did not differ across conditions.

Table S1: A summary of the BCEA analysis of fixation stability across the participants (n = 6). Each stimulus condition has one BCEA estimate that includes a size estimate and a center position of the BCEA ellipse.

|  | Conditions | Center | Full-Field | Filling-in | No Filling-in |
| --- | --- | --- | --- | --- | --- |
| Observer | BCEA parameter |  |  |  |  |
| P1^a^ | Size (in squared degree) | 0.98 | 1.02 | 1.03 | 0.91 |
|  | Center x, y position (in degree, relative to the fixation point at 0,0) | 0.46, 0.72 | 0.46, 0.44 | 0.43, 0.61 | 0.45, 0.64 |
| P2^a^ | Size (in squared degree) | 0.49 | 0.35 | 0.41 | 0.58 |
|  | Center x, y position (in degree, relative to the fixation point at 0,0) | 0.06, 0.06 | 0.10, 0.02 | -0.10, 0.13 | -0.10, 0.08 |
| P3^b^ | Size (in squared degree) | 23.99 | 21.51 | 15.66 | 16.55 |
|  | Center x, y position (in degree, relative to the fixation point at 0,0) | -6.58, 0.26 | -6.26, -0.12 | -6.57, 0.45 | -6.76, 0.37 |
| P4^b^ | Size (in squared degree) | 27.06 | 18.23 | 15.66 | 16.55 |
|  | Center x, y position (in degree, relative to the fixation point at 0,0) | 0.04, 2.38 | 0.24, 2.28 | 0.03, 2.57 | -0.10, 2.44 |
| P5^b^ | Size (in squared degree) | 39.69 | 39.49 | 36.40 | 38.14 |
|  | Center x position (in degree, relative to the fixation point at 0,0) | -3.49, 3.98 | -3.49, 4.01 | -3.47, 4.60 | -3.69, 4.74 |
| P6^b^ | Size (in squared degree) | 7.42 | 7.93 | 3.89 | 6.87 |
|  | Center x, y position (in degree, relative to the fixation point at 0,0) | -0.56, 4.20 | -0.54, 4.25 | -0.51, 4.27 | -0.66, 4.28 |

*Note.* a. Data recorded with the Eyelink 1000 eye tracker in the out-of-scanner control experiment; b. Data recorded with the MRI-safe ViewPoint eye tracker during the fMRI experiment.
